# Structural modeling of ion channels using AlphaFold2, RoseTTAFold2, and ESMFold

**DOI:** 10.1080/19336950.2024.2325032

**Published:** 2024-03-06

**Authors:** Phuong Tran Nguyen, Brandon John Harris, Diego Lopez Mateos, Adriana Hernández González, Adam Michael Murray, Vladimir Yarov-Yarovoy

**Affiliations:** a Department of Physiology and Membrane Biology, University of California School of Medicine, Davis, CA, USA; b Biophysics Graduate Group, University of California School of Medicine, Davis, CA, USA; c Monterey Peninsula College, Monterey, CA, USA; d Department of Anesthesiology and Pain Medicine, University of California School of Medicine, Davis, CA, USA

**Keywords:** Structural modeling, voltage-gated sodium channels, voltage-gated calcium channels, voltage-gated potassium chnanels, AlphaFold, RoseTTAFold, ESMFold

## Abstract

Ion channels play key roles in human physiology and are important targets in drug discovery. The atomic-scale structures of ion channels provide invaluable insights into a fundamental understanding of the molecular mechanisms of channel gating and modulation. Recent breakthroughs in deep learning-based computational methods, such as AlphaFold, RoseTTAFold, and ESMFold have transformed research in protein structure prediction and design. We review the application of AlphaFold, RoseTTAFold, and ESMFold to structural modeling of ion channels using representative voltage-gated ion channels, including human voltage-gated sodium (Na_V_) channel - Na_V_1.8, human voltage-gated calcium (Ca_V_) channel – Ca_V_1.1, and human voltage-gated potassium (K_V_) channel – K_V_1.3. We compared AlphaFold, RoseTTAFold, and ESMFold structural models of Na_V_1.8, Ca_V_1.1, and K_V_1.3 with corresponding cryo-EM structures to assess details of their similarities and differences. Our findings shed light on the strengths and limitations of the current state-of-the-art deep learning-based computational methods for modeling ion channel structures, offering valuable insights to guide their future applications for ion channel research.

## Introduction

Ion channels play key roles in human physiology and have been established as important targets in drug discovery [1,2]. The atomic-scale structures of ion channels provide invaluable insights into a fundamental understanding of the molecular mechanisms of channel gating and modulation. The recent advancements in cryo-electron microscopy (cryo-EM) produced a remarkable increase in the number of high-resolution structures of ion channels [3–7]. Multiple ion channel structures have been resolved in various putative physiological states and in complex with auxiliary subunits, small molecules, and natural peptides, providing crucial insights into the molecular mechanisms underlying their modulation.

In parallel to advancements in cryo-EM, breakthroughs in deep learning-based computational methods, such as AlphaFold [8] from Google’s DeepMind and RosetTTAFold [9,10] from David Baker’s Institute for Protein Design at the University of Washington, have been transforming research in protein structure prediction. These methods utilize deep neural networks trained on co-evolution information from multiple sequence alignments derived from protein sequence database (UniProt) [11] and protein structural data derived from Protein Data Bank (PDB) [12] to predict protein structures. AlphaFold and RoseTTAFold based methods have been applied to protein design and modeling of protein complexes [13–19]. Additionally, large language models of protein sequences, such as Meta AI’s ESMFold [20], trained on millions of protein sequences and using billions of parameters, provide rapid protein structure predictions, although with slightly lower accuracy compared to AlphaFold and RosettaFold. The ability to predict protein structures with high accuracy holds tremendous promise in transforming the field of drug discovery. Notably, the AlphaFold Structural Database currently contains over 200 million protein models predicted by AlphaFold [21] and the ESM Metagenomic Atlas contains more than 700 million protein models predicted by ESMFold [20].

Despite the remarkable achievements in protein structure prediction using deep-learning-based methods, the performance of these methods on challenging targets like ion channels remains to be determined. This is particularly significant when considering the structural heterogeneity of ion channel physiologically relevant states. To examine the structural modeling of ion channels using the deep-learning-based methods, we applied AlphaFold2 [8], RosetTTAFold2 [9], and ESMFold [20] to predict structures of representative voltage-gated ion channels, including human voltage-gated sodium (Na_V_) channel – Na_V_1.8, human voltage-gated calcium (Ca_V_) channel – Ca_V_1.1, and human voltage-gated potassium (K_V_) channel – K_V_1.3. We compared AlphaFold2, RoseTTAFold2, and ESMFold structural models of Na_V_1.8, Ca_V_1.1, and K_V_1.3 with corresponding cryo-EM structures of Na_V_1.8 [22], Ca_V_1.1 [23], and K_V_1.3 [24] to assess details of their similarities and differences. Our findings shed light on the strengths and limitations of the current state-of-the-art deep learning-based computational methods for modeling ion channel structures, offering valuable insights to guide their future applications for ion channel research.

## Structural modeling of voltage-gated sodium (Nav) channels

Voltage-gated sodium (Na_V_) channels are responsible for initiating and propagating action potentials, the electrical signals facilitating communication between excitable cells [1,25–27]. There are nine Na_V_ channel subtypes, from Na_V_1.1 to Na_V_1.9. The Na_V_1.1, Na_V_1.2, and Na_V_1.6 subtypes are predominantly expressed in the central nervous system [28]. The Na_V_1.4 and Na_V_1.5 subtypes are mainly expressed in skeletal and cardiac muscles, respectively [28]. The peripheral nervous system primarily expresses Na_V_1.7, Na_V_1.8, and Na_V_1.9 subtypes [28]. Dysfunctions in these channels can lead to serious health issues, including epilepsy, cardiac arrhythmias, muscle weakness, and chronic pain. The advancement of cryo-EM has facilitated the resolution of mammalian Na_V_ subtypes, ranging from Na_V_1.1 to Na_V_1.8, significantly enhancing our understanding of their structure, gating, and modulation [22,29–35].

The voltage-dependent gating, sodium conduction, and modulation by natural peptides and small molecule drugs are performed by the Na_V_ channel α subunit [1,25–27]. Auxiliary Na_V_ channel β subunits (β1-β4) are co-expressed with the α subunit and modulate the channel function [36]. We selected the Na_V_1.8 channel α subunit, as an example Na_V_ channel, for structure prediction using AlphaFold2 [8] and ColabFold as the computational platform [37]. ColabFold’s AlphaFold2 pipeline employs MMseqs2 multiple sequence alignment method [38,39], which is a more efficient alternative to Jackhmmer multiple sequence alignment method [40] used in DeepMind’s original AlphaFold2 pipeline [8]. The MMseqs2 method has considerably accelerated the AlphaFold2 protein structure prediction pipeline performance while maintaining comparable accuracy [37]. The protein sequence of the SCN10A gene, which encodes the human Na_V_1.8 (hNa_V_1.8) α subunit (UniProt ID: Q9Y5Y9), was used as input into ColabFold’s AlphaFold_mmseqs2 notebook for structure prediction. We assessed the quality of predicted AlphaFold2 models of hNa_V_1.8 using predicted local distance difference test (pLDDT) confidence score. Generally, pLDDT values above 90 represent very high confidence, pLDDT values between 70 and 90 represent good confidence, pLDDT values between 50 and 70 represent low confidence, and pLDDT values below 50 represent very low confidence [8]. We also compared similarities and differences to the resolved hNa_V_1.8 structures (PDB: 7WE4, 7WEL, 7WFR, and 7WFW) [22] using alpha carbon root mean square deviation (Cα RMSD). We assessed individual transmembrane voltage-sensing domains (VSD-I, VSD-II, VSD-III, and VSD-IV), the pore domain, the extracellular loop (ECL) regions, and the overall model topology.

**Figure 1.**
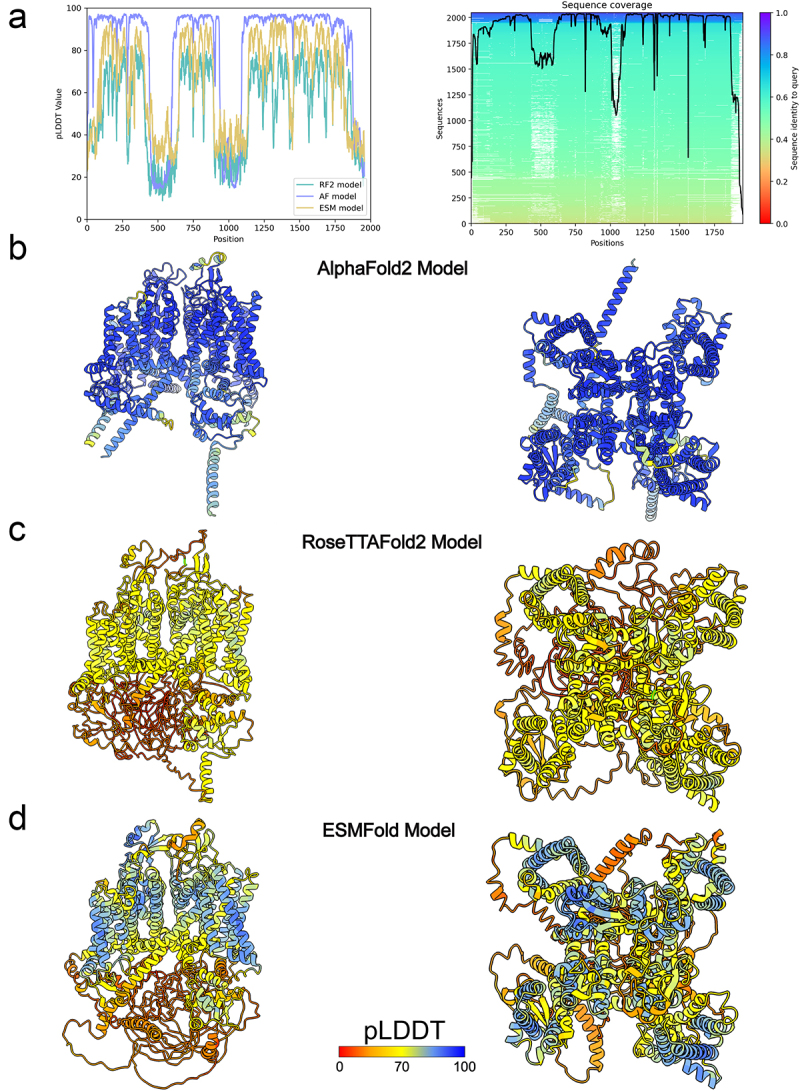
AlphaFold2, RoseTTAFold2, and ESMFold models of hNa_V_1.8. a) plot of pLDDT confidence score versus hNa_V_1.8 residue position for AlphaFold2 (AF), RoseTTAFold2 (RF2), and ESMFold (ESM) models. *right panel*, multiple sequence alignment of hNa_V_1.8 sequence and its homologs identified by MMseqs2 method [38,39] and used for AlphaFold modeling of hNa_V_1.8. a total number of homologous sequences identified per hNa_V_1.8 residue position is shown by a black trace. b) transmembrane (*left panel*) and extracellular (*right panel*) views of AlphaFold model of hNa_V_1.8. c) transmembrane (*left panel*) and extracellular (*right panel*) views of RoseTTAFold2 model of hNa_V_1.8. d) transmembrane (*left panel*) and extracellular (*right panel*) views of ESMFold model of hNa_V_1.8. AlphaFold2, RoseTTAFold2, and ESMFold models are colored by confidence score (pLDDT) from very low confidence (red) to good confidence (yellow) to high confidence (blue).

Our results showed that AlphaFold2 could predict the majority of the hNa_V_1.8 domains with very high confidence scores (pLDDT >90), ESMFold could predict VSDs, the pore domain, and ECL regions with good confidence (70 < pLDDT < 90), while RoseTTAFold2 predicted most transmembrane regions with low confidence (50 < pLDDT < 70) and predicted the pore domain with good confidence (Figure 1).

**Figure 2.**
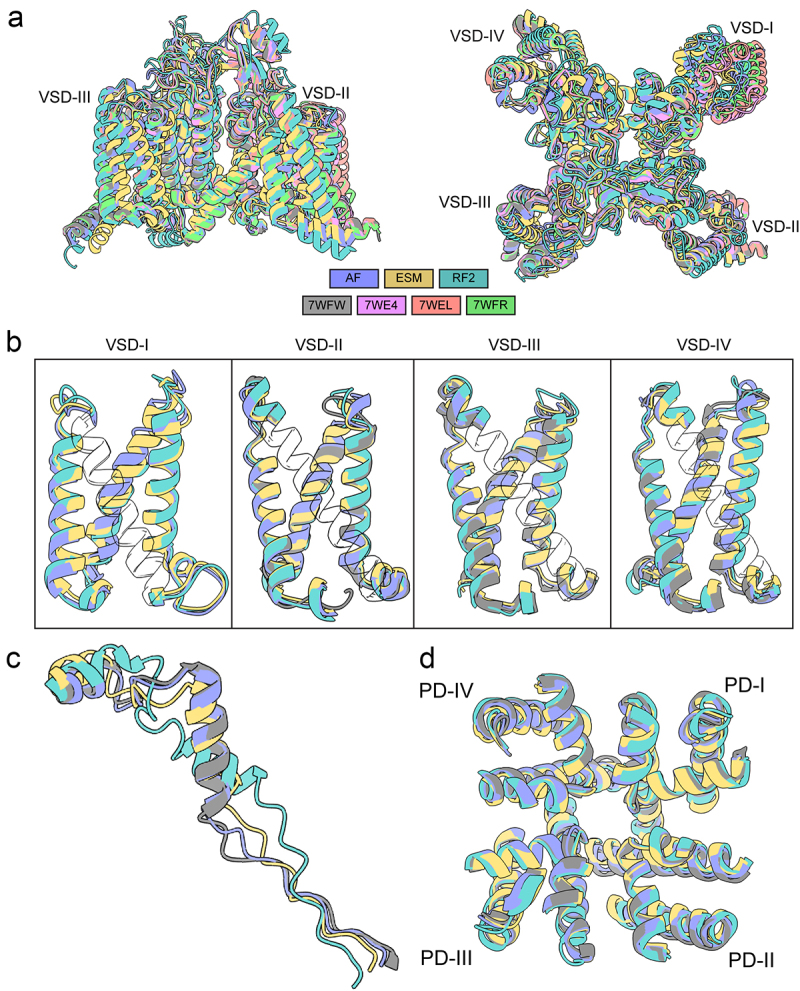
Comparison of AlphaFold2, RoseTTAFold2, and ESMFold models and cryoEM structures of hNa_V_1.8. a) transmembrane (*left panel*) and extracellular (*right panel*) views of AlphaFold2, RoseTTAFold2, and ESMFold models superimposed to cryoEM structures of hNa_V_1.8 (PDB: 7WE4, 7WEL, 7WFR, and 7WFW) [22]. Intrinsically disordered *N*- and C-termini predicted by AlphaFold2, RoseTTAFold2, and ESMFold with low confidence were removed for clarity. b) transmembrane view of AlphaFold2, RoseTTAFold2, and ESMFold VSD-I, VSD-II, VSD-III, and VSD-IV models superimposed to apo-state hNa_V_1.8 structure (PDB: 7WFW). c) extracellular view of AlphaFold2, RoseTTAFold2, and ESMFold domain III-IV intracellular linker models superimposed to apo-state hNa_V_1.8 structure. d) extracellular view of AlphaFold2, RoseTTAFold2, and ESMFold pore domain (PD) models superimposed to apo-state hNa_V_1.8 structure.

Relative to published structures, the overall topology of the models closest resembles the apo state (PDB: 7WFW), with AlphaFold2 having the lowest Cα RMSD at 2.0 Å (Figure 2(a)). This is exemplified by the AlphaFold2 model being able to predict VSD-II, VSD-II, VSD-IV (Figure 2(b)), and the domain III-IV intracellular linker (Figure 2(c)) with less than 2.0 Å Cα RMSD to the apo state structure (VSD-I is not resolved in the apo state). Notably, all methods produced pore domain Cα RMSD 2.0 Å or less relative to the apo-state, with AlphaFold2 having the lowest Cα RMSD at 0.72 Å (Figure 2(d)).

For all three methods, the N-terminal domain (NTD) residues from M1 to N11, domain I-II intracellular loop region residues from L442 to D602, domain II-III intracellular loop region residues from S935 to L1092, and C-terminal domain (CTD) residues from N1875 to P1956 have low confidence scores (Figure 1). The low prediction confidence scores suggest that these regions are either highly flexible or inherently disordered in the absence of interacting intracellular partners. Notably, AlphaFold2 also predicted with good and high confidence scores parts of the NTD preceding the VSD-I (residues from N12 to S130), domain II-III intracellular loop region residues from N908 to R934, domain III-IV intracellular loop region from K1427 to N1463 that contains the intracellular gate important for Na_V_ channel fast inactivation, and segments of the CTD (residues from T1733 to S1874). The NTD, domain I-II intracellular loop region, domain I-III intracellular loop region, and CTD regions are absent in currently resolved structures of hNa_V_1.8 (PDB IDs: 7WE4, 7WEL, 7WFR, and 7WFW) [22] but have been resolved in cryo-EM structures of other Na_V_ channel subtypes [22,29–35]. The domain III-IV intracellular linker residues from K1427 to N1463 consistently had the closest match between hNa_V_1.8 structures and AlphaFold2 models (Cα RMSD = 1.1 Å), compared to ESMFold models (Cα RMSD = 2.6–2.8 Å) and RoseTTAFold2 models (Cα RMSD = 5.5–5.6 Å) (Figure 2(c)).

**Figure 3.**
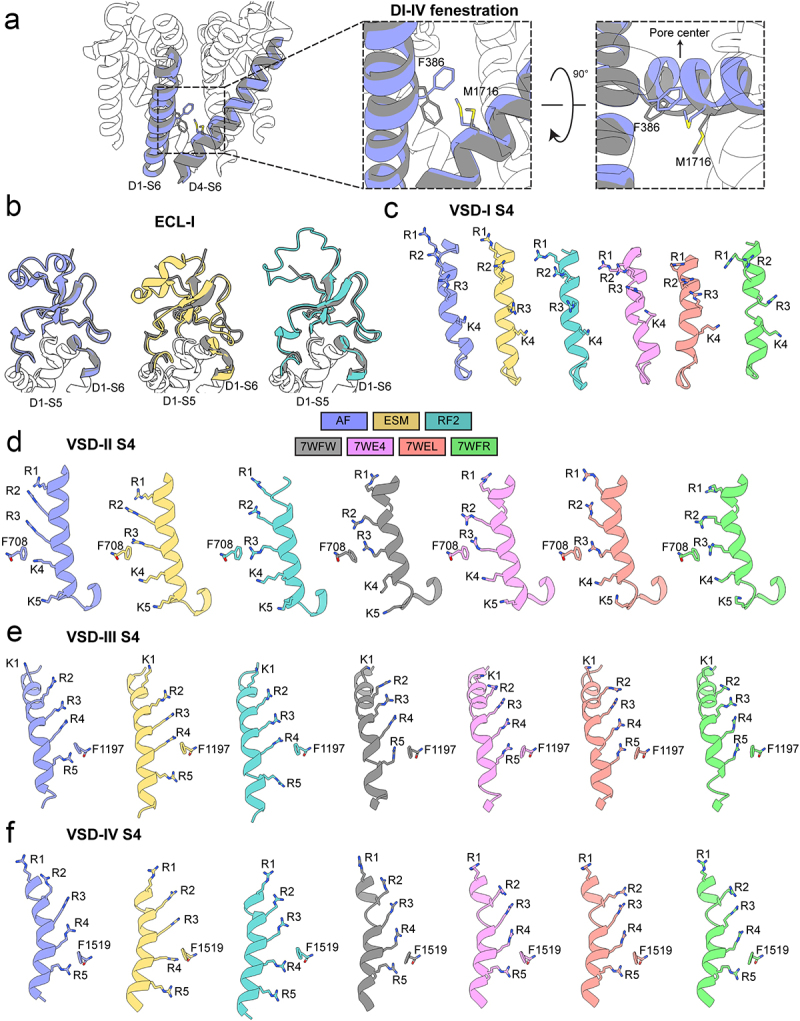
Comparison of specific regions in AlphaFold2, RoseTTAFold2, and ESMFold models to cryoEM structures of hNa_V_1.8. a) transmembrane (*left and middle panel*), and extracellular (*right panel*) views of F386 relative to M1716 at the domains I-IV (DI-IV) interface fenestration region in AlphaFiold2 model superimposed to apo-state hNa_V_1.8 structure (PDB: 7WFW). b) comparison of AlphaFold2, RoseTTAFold2, and ESMFold extracellular loop in domain I (ECL-I) model predictions relative to the partially resolved ECL-I in the apo-state hNa_V_1.8 structure. c) comparison of VSD-I S4 gating charges in AlphaFold2, RoseTTAFold2, and ESMFold models relative to cryoEM structures of hNa_V_1.8 (PDB: 7WE4, 7WEL, and 7WFR) [22]. d) comparison of VSD-II S4 gating charges in AlphaFold2, RoseTTAFold2, and ESMFold models relative to cryoEM structures of hNa_V_1.8 (PDB: 7WE4, 7WEL, 7WFR, and 7WFW) [22]. e) comparison of VSD-III S4 gating charges in AlphaFold2, RoseTTAFold2, and ESMFold models relative to cryoEM structures of hNa_V_1.8 (PDB: 7WE4, 7WEL, 7WFR, and 7WFW) [22]. f) comparison of VSD-IV S4 gating charges in AlphaFold2, RoseTTAFold2, and ESMFold models relative to cryoEM structures of hNa_V_1.8 (PDB: 7WE4, 7WEL, 7WFR, and 7WFW) [22]. Side chains of gating charge-carrying residues in the S4 segments are shown in stick representation and labeled.

Focusing on the pore region, the AlphaFold2, ESMFold, and RoseTTAFold2 models of hNa_V_1.8 align closely with the experimentally resolved structures of hNa_V_1.8 (Cα RMSD = 0.7–1.8 Å) Notably, the AlphaFold2 model exhibits only minor differences over the selectivity filter, P1-helix, P2-helix, S5, and S6 segments (Figure 2(d)). One noticeable difference is the conformation of domain I S6 segment (DI-S6). AlphaFold2’s model shows a slight deviation in the helical turn near F386 residue in DI-S6, causing its side chain to point toward the DI-IV fenestration and pack together with M1716 in DIV-S6 (Figure 3(a)). In contrast, the cryo-EM structures of hNa_V_1.8 resolved in complex with a small molecule-based compound (A-803467) and in the apo state show F386 pointing downwards and M1716 oriented away from the fenestration. This difference between the AlphaFold2 model and the cryo-EM structure of hNa_V_1.8 may arise due to a different conformation of DI-S6 captured in the AlphaFold2 model. Interestingly, AlphaFold2 predicted a conformation of the ECL in domain I (ECL-I) with high confidence, which was partially unresolved in the cryo-EM structures of hNa_V_1.8. Specifically, residues 279 to 282 and 289 to 297 in ECL-I adopted a helical conformation with high confidence in the AlphaFold2 model of hNa_V_1.8 but are unstructured in the cryo-EM structure of hNa_V_1.8 (Figure 3(a)). For the same residues in ECL-I, ESMFold adopts a helical conformation between residues 283 to 291 with very low confidence (pLDDT <50), and RoseTTAFold2 adopts a loop conformation between residues 279 to 297 with very low confidence (Figure 3(a)).

The voltage sensor domains were predicted with high confidence in the AlphaFold2 model, good confidence in the ESMFold model, and low confidence in the RoseTTAFold2 model of hNa_V_1.8. However, the S3-S4 loop regions in VSD-I and VSD-II were predicted with good confidence in AlphaFold2, low confidence in ESMFold, and very low confidence in RoseTTAFold2, reflecting their flexibility (Figure 1). Despite the high confidence in the VSD predictions, comparison with the experimentally resolved hNa_V_1.8 structures revealed several key differences. The cryo-EM structures of hNa_V_1.8 in complex with A-803467 revealed various conformations of VSD-I, represented by class I, II, and III structures (PDB IDs: 7WE4, 7WEL, and 7WFR) [22]. VSD-I was not resolved in the cryo-EM structures of hNa_V_1.8 in an apo state (PDB: 7WFW) [22]. However, conformations of VSD-I in the AlphaFold2, ESMFold, and RoseTTAFold2 models of hNa_V_1.8 don’t align with the conformation of VSD-I in any of the cryo-EM structures of hNa_V_1.8 (Figure 3(c)) (Cα RMSD = 2.5–3.8 Å). With a very high pLDDT confidence score, this discrepancy raises a possibility that AlphaFold2’s model of hNa_V_1.8 represents another apo state of VSD-I. The AlphaFold2 model of hNa_V_1.8 VSD-II appears to be in a fully “up” state, with the gating charges R1, R2, and R3 in the S4 segment of VSD-II positioned above the gating charge transfer residue F708 in the S2 segment of VSD-II. In contrast, the class I cryo-EM structure of hNa_V_1.8 in complex with A-803467 (PDB: 7WE4) shows the gating charge R3 in the S4 segment of VSD-II at the gating charge transfer, considered to be a “half-click” down from the VSD-II state observed in the AlphaFold2 model (see Figure 2(b)). These observations may explain the difference in S3-S4 region conformations in VSD-II between the hNa_V_1.8 structures and AlphaFold2 models (Figure 3(d)).

In contrast to the models of hNa_V_1.8 VSD-I and VSD-II, AlphaFold2 conformations of VSD-III and VSD-IV appear to align closely with conformations of corresponding VSD-III and VSD-IV in the cryo-EM structures of hNa_V_1.8 (VSD-III: Cα RMSD = 0.6–0.8 Å, VSD-IV: Cα RMSD = 1.2–1.3 Å) (Figure 2(b)). ESMFold and RoseTTAFold2 both performed worse in comparison to AlphaFold2 (ESMFold VSD-III: Cα RMSD = 1.5 Å, ESMFold VSD-IV: Cα RMSD = 1.8–1.9 Å, RoseTTAFold2 VSD-III: Cα RMSD = 1.8–1.9 Å, RoseTTAFold2 VSD-IV: Cα RMSD = 2.4 Å). The backbone conformations of VSD-III in the AlphaFold2 model almost identically match those in the resolved structure (PDB: 7WE4), with gating charges consistently at the same position relative to the gating transfer (Figure 3(e)). Similarly, the gating charges in the S4 segment of VSD-IV in the AlphaFold2 models and cryo-EM structures of hNa_V_1.8 also occupy similar positions (Figure 3(f)). However, the S3-S4 conformations in VSD-IV are significantly different between the AlphaFold2 models and cryo-EM structures of hNa_V_1.8. As with VSD-I, there is heterogeneity observed in the S3-S4 region of VSD-IV, with multiple conformations resolved in cryo-EM structures of hNa_V_1.8 (PDB: 7WE4, 7WEL, and 7WFR, and 7WFW) [22]. Similar to the observations with VSD-II, the high confidence prediction of the VSD-IV S3-S4 region conformations by AlphaFold2 suggests a potentially different conformation of this region of hNa_V_1.8.

## Structural modeling of voltage-gated calcium (Cav) channels

Voltage-gated calcium (Ca_V_) channels mediate Ca^2+^ influx upon depolarization of cell membrane potentials [1,2,25,41,42]. Ten subtypes of Ca_V_ channels are divided into three main subfamilies, Ca_V_1, Ca_V_2, and Ca_V_3, each serving distinct and crucial roles in physiological functions [28]. The Ca_V_1 channels are responsible for muscle contraction, hormone secretion, and integrating synaptic inputs. The Ca_V_2 channels play a key role in rapid communication in nerve cells. The Ca_V_3 channels are crucial for the repetitive firing of action potentials in rhythmically firing cells, such as cardiac myocytes and thalamic neurons, contributing to regulating heart rhythm and synchronizing neural activities.

Cav1.1 structure is composed of α1, α2, β, *γ*, and *γ* subunits with α1 subunit responsible for voltage-dependent gating, calcium conduction, and modulation by small molecule drugs [23,43,44]. The protein sequence of CACNA1S gene encoded human Ca_V_1.1 (hCa_V_1.1) α subunit (UniProt ID: Q13698) was used as input into AlphaFold2, RoseTTAFold2, and ESMFold for structure prediction. We assessed the quality of predicted models using the pLDDT confidence score, compared how close they are to the resolved rabbit Ca_V_1.1 structure (rCa_V_1.1) (PDB: 5GJV) [43], and discussed the structural variations.

**Figure 4.**
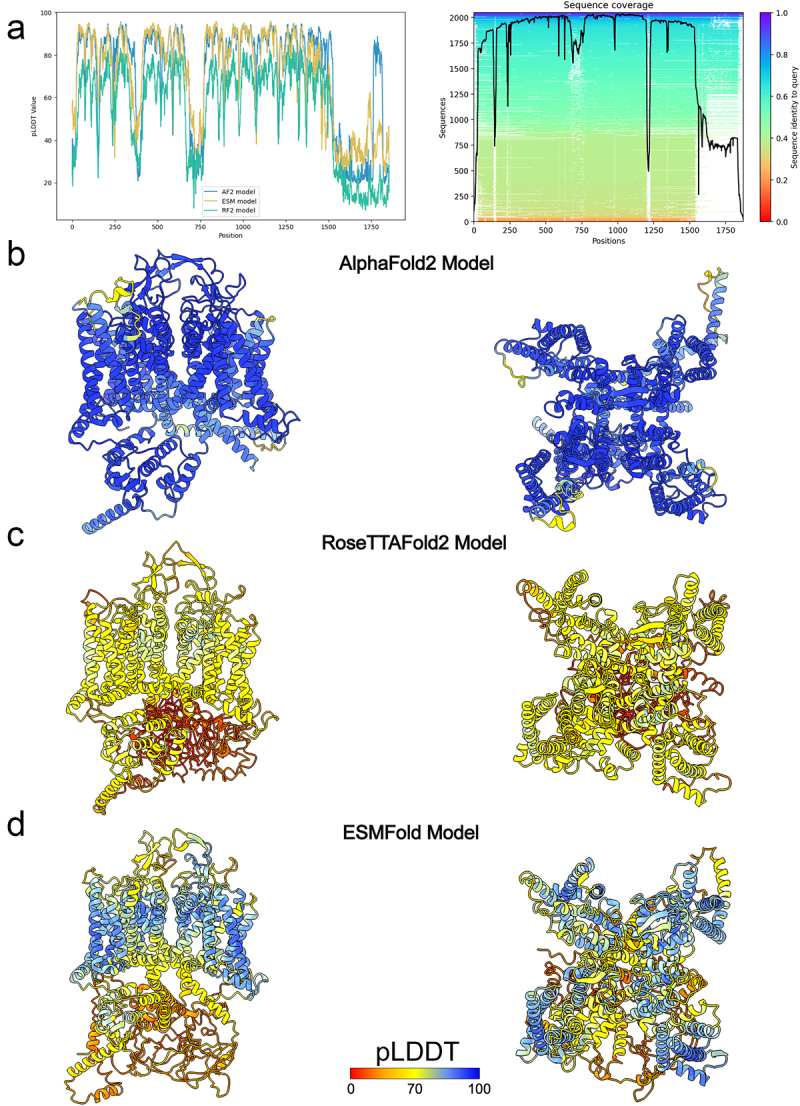
AlphaFold2, RoseTTAFold2, and ESMFold models of hCa_V_1.1. a) plot of pLDDT confidence score versus hCa_V_1.1 residue position for AlphaFold2 (AF), RoseTTAFold2 (RF2), and ESMFold (ESM) models. *right panel*, multiple sequence alignment of hCa_V_1.1 sequence and its homologs identified by MMseqs2 method [38,39] and used for AlphaFold modeling of hCa_V_1.1. a total number of homologous sequences identified per hCa_V_1.1 residue position is shown by a black trace. b) transmembrane (*left panel*) and extracellular (*right panel*) views of AlphaFold model of hCa_V_1.1. c) transmembrane (*left panel*) and extracellular (*right panel*) views of RoseTTAFold2 model of hCa_V_1.1. d) transmembrane (*left panel*) and extracellular (*right panel*) views of ESMFold model of hCa_V_1.1. AlphaFold2, RoseTTAFold2, and ESMFold models are colored by confidence score (pLDDT) from very low confidence (red) to good confidence (yellow) to high confidence (blue).

**Figure 5.**
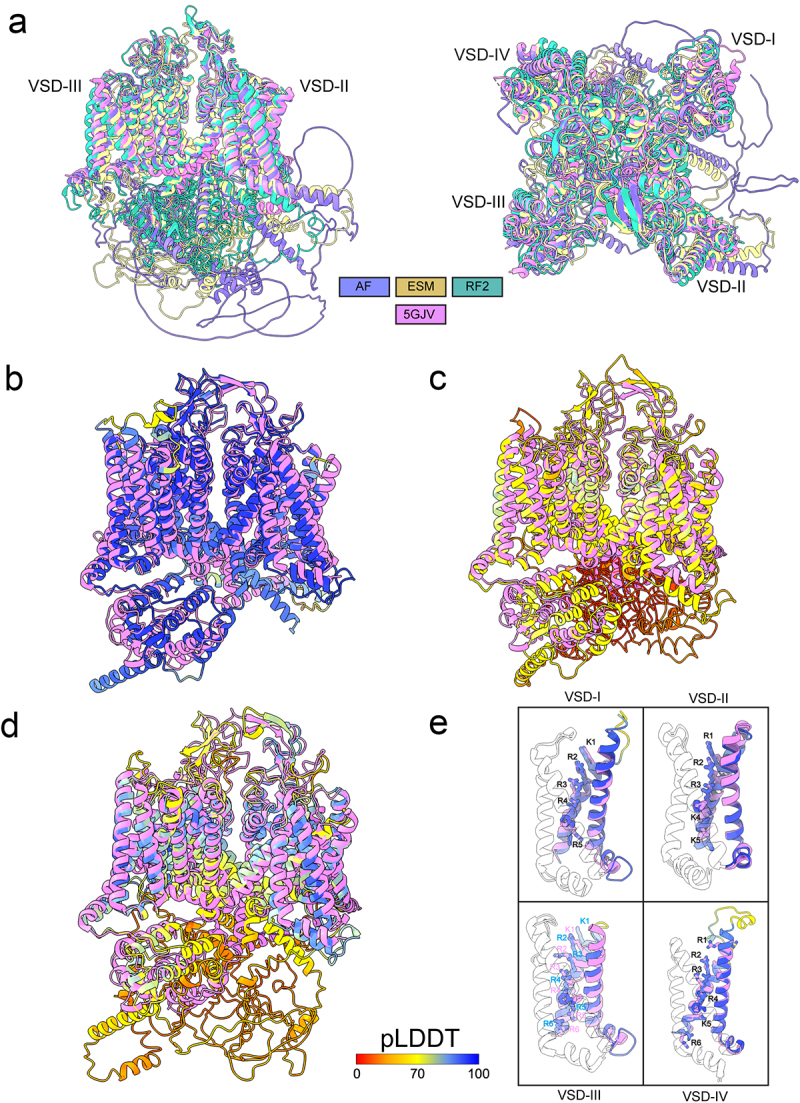
Comparison of AlphaFold2, RoseTTAFold2, and ESMFold models and cryoEM structures of hCa_V_1.1. a) Transmembrane (*left panel*) and extracellular (*right panel*) views of AlphaFold2, RoseTTAFold2, and ESMFold models superimposed to cryoEM structure of hCa_V_1.1 (PDB: 5GJV). b) transmembrane view of AlphaFold2 model of hCa_V_1.1 superimposed to cryoEM structure of hCa_V_1.1 (PDB: 5GJV). c) transmembrane view of RoseTTAFold2 model of hCa_V_1.1 superimposed to cryoEM structure of hCa_V_1.1 (PDB: 5GJV). d) transmembrane view of ESMFold model of hCa_V_1.1 superimposed to cryoEM structure of hCa_V_1.1 (PDB: 5GJV). AlphaFold2, RoseTTAFold2, and ESMFold models are colored by confidence score (pLDDT) from very low confidence (red) to good confidence (yellow) to high confidence (blue). e) transmembrane view of AlphaFold model of hCa_V_1.1 VSD-I, VSD-II, VSD-III, and VSD-IV colored in blue superimposed with cryoEM structures of hCa_V_1.1 (PDB: 5GJV) colored in purple. Side chains of gating charge-carrying residues in the S4 segments are shown in stick representation and labeled.

Our results showed that most of hCa_V_1.1 structure was predicted with high confidence by AlphaFold2 (overall pLDDT = 71.4) and ESMFold (overall pLDDT = 70.6) and with low confidence by RoseTTAFold2 (overall pLDDT = 54.1) (Figures 4 and 5). Specifically, hCa_V_1.1 VSD-I, VSD-II, VSD-III, and VSD-IV, pore domain, and ECL regions are predicted with high confidence (pLDDT >90) by AlphaFold2 and ESMFold and with good confidence by RoseTTAFold2 (60 < pLDDT < 90). However, most of the N-terminal domain formed by residues from M1 to K18, part of domain I-II intracellular loop region residues from L386 to A402, part of domain II-III intracellular loop region residues from L705 to E768, and most of the CTD regions formed by residues from Y1546 to S1780 and from G1835 to L1837 have been predicted with low confidence (pLDDT <50) by AlphaFold2, ESMFold, and RoseTTAFold2 (Figure 4(a)). This low confidence prediction suggests that these regions are either highly flexible or inherently disordered in the absence of interacting intracellular partners (Figure 4(a)). Interestingly, AlphaFold2 also predicted parts of the NTD from P19 to K51, part of domain I-II intracellular loop region residues from R347 to K385, part of domain II-III intracellular loop region residues from G689 to K704, domain III-IV intracellular loop region residues from G689 to K704 from E1073 to P1106, and CTD region residues from N1383 to G1545 with good confidence (70 < pLDDT < 90) and high confidence (pLDDT >90) (Figure 4(a,b)).

In the AlphaFold2, ESMFold, and RoseTTAFold2 models of hCa_V_1.1, the extracellular regions of the pore relatively closely match the cryo-EM structure of rCa_V_1.1 [43], exhibiting only minor differences over the selectivity filter, P1-helix, P2-helix, and ECL regions (Cα RMSD = 1.09 Å) (Figure 5(a-d)). However, conformations of the intracellular half of the S6 segments are captured in a different state in the AlphaFold2, ESMFold, and RoseTTAFold2 model of hCa_V_1.1 compared to the cryo-EM structure of rCa_V_1.1 (Cα RMSD = 4.96 Å) (Figure 5(a-d)).

The voltage sensor domains were predicted with high confidence (pLDDT >90) in the AlphaFold2 model of hCa_V_1.1. However, a significant drop in confidence was observed in the S3-S4 regions, reflecting their higher flexibility (Figure 5(b,e)). Notably, VSD-I state in the AlphaFold2 model of hCa_V_1.1 is matching closely VSD-I state in the cryo-EM structure of rCa_V_1.1 (Figure 5(e)). Similarly, the gating charges in the S4 segment of VSD-I in the AlphaFold2 model of hCa_V_1.1 and cryo-EM structures of rCa_V_1.1 also occupy similar positions (Figure 5(e)). VSD-II state in the AlphaFold2 model of hCa_V_1.1 has the S4 segment positioned a “half-click” down compared to VSD-II state in the cryo-EM structure of rCa_V_1.1 (Figure 5(e)). VSD-III state in AlphaFold2 model of hCa_V_1.1 has the S4 segment positioned a “half-click” up compared to VSD-III state in the cryo-EM structure of rCa_V_1.1 (Figure 5(e)). VSD-IV state in AlphaFold2 model of hCa_V_1.1 matches closely VSD-IV state in the cryo-EM structure of rCa_V_1.1 (Figure 5(e)). Similarly, the gating charges in the S4 segment of VSD-IV in the AlphaFold2 model of hCa_V_1.1 and cryo-EM structures of rCa_V_1.1 also occupy similar positions. The S3-S4 loop regions in VSD-I, VSD-III, and VSD-IV have not been resolved in the cryo-EM structure of rCa_V_1.1 (PDB: 5GJV) [43]. Notably, the S3-S4 loop regions in VSD-I, VSD-III, and VSD-IV AlphaFold2 models of hCa_V_1.1 have good confidence (pLDDT >70) (Figure 5(e)).

## Structural modeling of voltage-gated potassium (Kv) channels

Voltage-gated potassium (K_V_) channels mediate K^+^ efflux upon membrane depolarization and regulate membrane potential [1,2]. There are 40 subtypes of K_V_ channels divided into 12 main subfamilies, from K_V_1 to K_V_12 [28]. Differently from Na_V_ and Ca_V_ channels, K_V_ channels are tetramers where each VSD-PD pair forms a separate subunit. The K_V_1.3 channels regulate membrane potential and calcium signaling in lymphocytes and oligodendrocytes [45]. These channels form homotetramers with the VSDs and PDs forming the membrane-spanning region and a cytosolic tetramerization (T1) domain [24]. The protein sequence of KCNA3 gene encoded human K_V_1.3 (hK_V_1.3) (UniProt ID: P22001) was used as input into AlphaFold2, RoseTTAFold2, and ESMFold for structure prediction. We assessed the quality of predicted models using the pLDDT confidence score, compared how close they are to the resolved hK_V_1.3 structures (PDB: 7SSX and 7SSY) [24], and discussed the structural variations.

**Figure 6.**
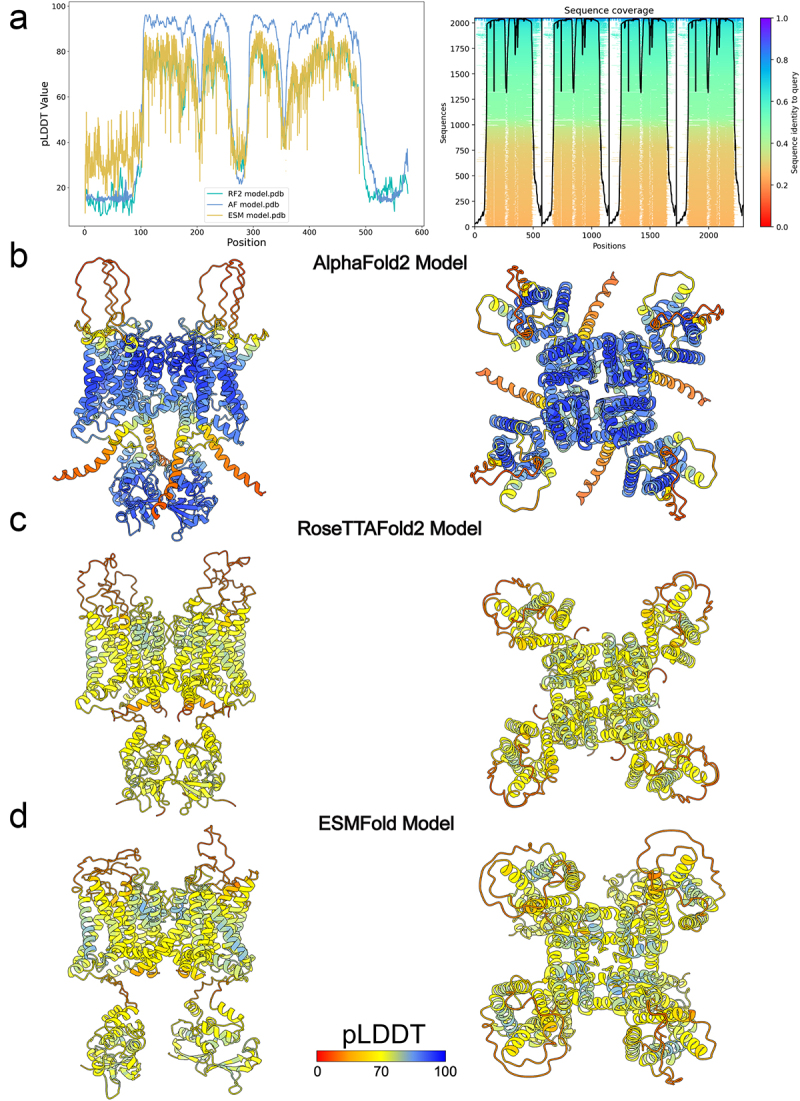
AlphaFold2, RoseTTAFold2, and ESMFold models of hK_V_1.3. a) plot of pLDDT confidence score versus hK_V_1.3 residue position for AlphaFold2 (AF), RoseTTAFold2 (RF2), and ESMFold (ESM) models. *right panel*, multiple sequence alignment of hK_V_1.3 sequence and its homologs identified by MMseqs2 method [38,39] and used for AlphaFold modeling of hK_V_1.3. a total number of homologous sequences identified per hK_V_1.3 residue position is shown by a black trace. b) transmembrane (*left panel*) and extracellular (*right panel*) views of AlphaFold model of hK_V_1.3. c) transmembrane (*left panel*) and extracellular (*right panel*) views of RoseTTAFold2 model of hK_V_1.3. d) transmembrane (*left panel*) and extracellular (*right panel*) views of ESMFold model of hK_V_1.3. Unstructured *N*- and C- terminals are not shown for clarity in AlphaFold, RoseTTAFold2 and ESMFold models. AlphaFold2, RoseTTAFold2, and ESMFold models are colored by confidence score (pLDDT) from very low confidence (red) to good confidence (yellow) to high confidence (blue).

**Figure 7.**
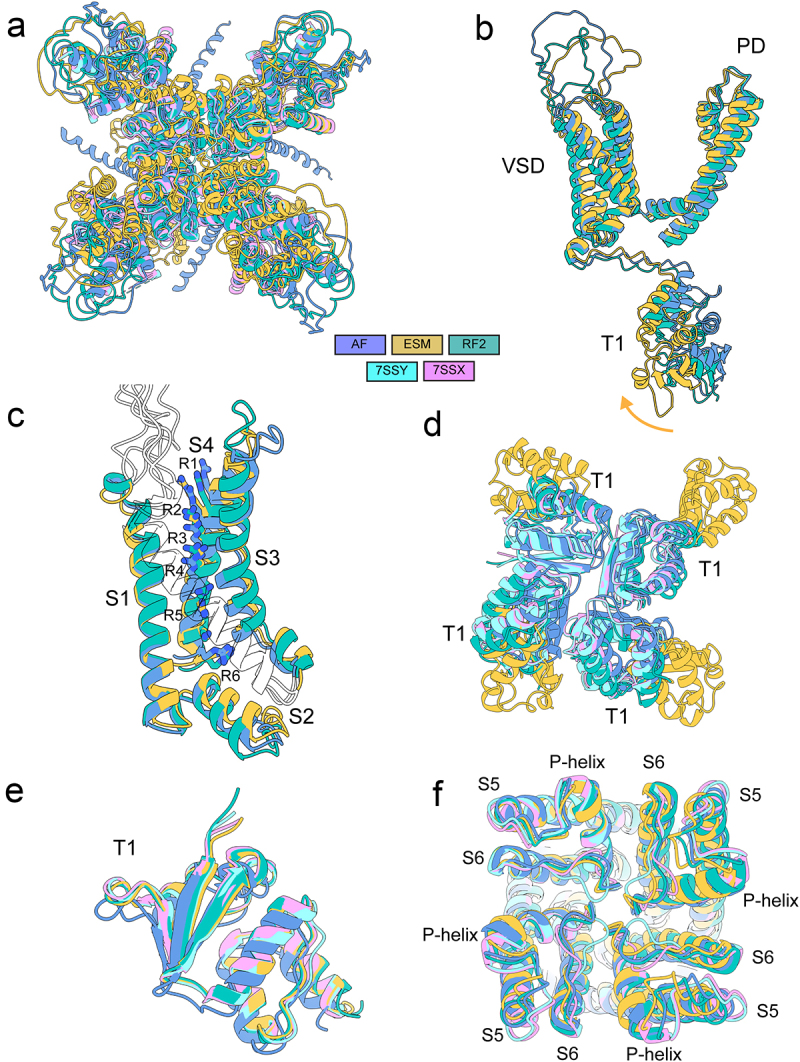
Comparison of specific regions in AlphaFold2, RoseTTAFold2, and ESMFold models to cryoEM structures of hK_V_1.3. a) extracellular view of AlphaFold2, RoseTTAFold2, and ESMFold models of hK_V_1.3 superimposed with cryoEM structures of hK_V_1.3 (PDBs: 7SSX and 7SSY). b) superimposition of AlphaFold, RoseTTAFold2, and ESMFold models. The yellow arrow indicates the tilt observed in the ESMFold model of the T1 domain. c) transmembrane view of AlphaFold, RoseTTAFold2, and ESMFold models of hK_V_1.3 VSD. The side chains of the gating charges located in the S4 segment are shown in stick representation and labeled. d) intracellular view of AlphaFold2, RoseTTAFold2, and ESMFold models of hK_V_1.3 T1 domain after superimposition of the full models with the cryoEM structures of hK_V_1.3 colored in purple (PDB: 7SSX) and cyan (PDB: 7SSY). e) superimposition of individual T1 domains from AlphaFold2, RoseTTAFold2, and ESMFold models with the T1 domains from cryoEM structures of hK_V_1.3. f) extracellular view of AlphaFold2, RoseTTAFold2, and ESMFold models of hK_V_1.3 pore domain superimposed with cryoEM structures of hK_V_1.3 pore.

Our results showed that AlphaFold2 predicted the transmembrane region of hK_V_1.3 formed by VSDs and pore domain with high confidence (pLDDT >90), as illustrated in Figures 6 and 7. The first part of the N-terminal region formed by residues from M1 to E104 was predicted with low confidence (pLDDT <50) (Figure 6(a)). The second part of the N-terminal region that comprises the T1 domain formed by residues from R105 to S230 was predicted with good confidence (70 < pLDDT < 90) or high confidence (pLDDT >90) and closely matched cryo-EM structures of hK_V_1.3 [24] N-terminal region formed by the same region (Cα RMSD = 1.4 Å) (Figure 6(a)). All of the pore region formed by residues from M395 to T491 was predicted with good confidence (70 < pLDDT < 90) or high confidence (pLDDT >90) and closely matched cryo-EM structure of hK_V_1.3 [24] pore region (PDB: 7SSX) (Cα RMSD = 1.4 Å) (Figure 6(a,b)). C-terminal region formed by residues from E492 to V575 has low confidence (pLDDT <50) (Figure 6(a)). This low prediction confidence suggests that this region is either highly flexible or inherently disordered in the absence of interacting partners. Most of the VSD region formed by residues from P232 to S381 was predicted with good confidence (70 < pLDDT < 90) or high confidence (pLDDT >90) and closely matched cryo-EM structure of hK_V_1.3 [24] VSD region (Cα RMSD = 1.9 Å) (Figures 6(a) and 7(c)). However, the S1-S2 and S3-S4 loop regions were predicted with low confidence (pLDDT <50), reflecting unstructured nature of these regions that have not been resolved in the cryo-EM structures of hK_V_1.3 [24] (Figure 7(c)). The relative position of the gating charges in the S4 segment revealed a similar state for the modeled VSD as the one observed in the cryo-EM experimental structures (Figure 7(c)).

RoseTTAFold2 prediction for K_V_1.3 showed the expected architecture with the transmembrane domains, building up the VSDs and PDs, and the intracellular T1 domain (Figure 6(a)). The confidence of the prediction was good at the well-structured regions (80 < pLDDT < 90), but lower compared to the AF models, while it presented low confidence (pLDDT <50) at the N and C terminal regions, as well as at the S1-S2 and S3-S4 unstructured loops of the VSDs (Figure 6(a,c)). ESMFold prediction resulted in a similar model that also had good confidence prediction (80 < pLDDT < 90) at the well-structured transmembrane domains and T1 domain, and low (pLDDT <50) at the N and C terminal regions and VSD extracellular loops (Figures 6(a,d)). The predicted state of the VSDs was similar to the one in the AF models (Figure 7(c)).

Notably, the ESMFold model of K_V_1.3 presented a different arrangement of the T1 domains, in which the domain independently has the expected fold when compared to the available cryo-EM structures (Figure 7(e)) (RMSD <1 Å), but the domain of each subunit shifts outwards (Figure 7(b), yellow arrow) breaking the interactions among the four subunit T1 domains that are observed in AlphaFold2 and RoseTTAFold2 models and solved structures (Figure 7(d)). The predicted structure for the K_V_1.3 pore in AlphaFold2, RoseTTAFold2, and ESMFold models closely matched the one observed in experimental structures (RMSD = 1.5 Å) (Figure 7(f)).

## Conclusions

Deep learning-based methods, such as AlphaFold [8], RosetTTAFold [9,10], and ESMFold [20] are useful for predicting structures of transmembrane regions of ion channels, including the voltage-sensing and pore domains, with high confidence. The extracellular and intracellular loop regions and intracellular *N*- and C-termini regions can be potentially predicted with high confidence if they are formed by α-helical or β-sheet secondary structure. Deep learning-based methods may predict alternative conformations of ion channels compared to known structures of identical or homologous ion channels. However, the accuracy of alternative ion channel conformations is only determined once confirmed by structural and experimental data. Modeling unstructured extracellular and intracellular loop regions and intracellular *N*- and C-termini regions remains challenging in the absence of potential protein partners to stabilize specific conformations of these regions. Structure prediction of ion channels using deep learning-based methods might be useful for designing therapeutics and molecular probes targeting specific ion channel subtypes. Finally, structural modeling of ion channels in complex with other proteins deep learning-based methods might reveal molecular mechanisms of ion channel modulation by extracellular, transmembrane, and intracellular proteins.

## Data Availability

AlphaFold2, ESMFold, and RoseTTAFold2 models of hNaV1.8, hCaV1.1, and hKV1.3 are available for download through the DRYAD database (https://doi.org/10.5061/dryad.z08kprrn0).
